# Mortality of People With Alzheimer's Disease and Psychiatric Morbidity: A Nationwide Finnish Cohort Study

**DOI:** 10.1002/gps.70210

**Published:** 2026-04-10

**Authors:** Aleksi Alastalo, Marianne Haapea, Tanja Nordström, Anna‐Maija Tolppanen, Jouko Miettunen, Miika Nietola, Sirpa Hartikainen, Erika Jääskeläinen

**Affiliations:** ^1^ Research Unit of Population Health University of Oulu Oulu Finland; ^2^ Medical Research Center Oulu Oulu University Hospital and University of Oulu Oulu Finland; ^3^ School of Pharmacy University of Eastern Finland Kuopio Finland; ^4^ Department of Psychiatry Oulu University Hospital Wellbeing Services County of North Ostrobothnia Oulu Finland; ^5^ Department of Psychiatry University of Turku Turku Finland; ^6^ Turku University Hospital Turku Finland

**Keywords:** Alzheimer's disease, cause of death, mortality, psychiatric comorbidity, psychiatric disorder

## Abstract

**Objectives:**

Psychiatric comorbidities are common in Alzheimer's disease (AD), and they have a negative impact on quality of life, but their impact on mortality remains unclear. This study examined mortality among persons with AD and psychiatric morbidity compared to persons with AD without psychiatric morbidity. This is the first study to analyze Alzheimer's disease mortality based on the timing of earlier psychiatric morbidity.

**Methods:**

We utilized the nationwide register‐based MEDALZ cohort, including 70,718 Finnish individuals diagnosed with AD between 2005 and 2011. Individuals were categorized into four groups based on the occurrence of their hospital‐treated psychiatric diagnosis before the diagnosis of AD. Mortality was assessed over an 8‐year follow‐up. Chi‐square test was used to compare the differences between the four groups in terms of causes of death and annual cumulative mortality. We determined cumulative mortality curves and hazard ratios, stratified by age, gender, cardiovascular disease, and the year of AD diagnosis.

**Results:**

During the 8‐year follow‐up, 70.4% of the AD cohort had died. Persons with earlier psychiatric morbidity had an AD diagnosis 1.4–4.3 years earlier and died 1.4–4.1 years younger. Mortality risk was slightly higher among those with psychiatric morbidity compared to those without (adjusted HRs 1.03–1.41), with the effect decreasing over the years of follow‐up. Mortality risk was not affected by the timing of psychiatric morbidity.

**Conclusion:**

Psychiatric comorbidity is associated with earlier AD onset and reduced lifespan; however, post‐diagnosis survival appears to be largely determined by AD progression itself.

## Key Points

- People with Alzheimer's disease and prior psychiatric hospitalization are diagnosed with Alzheimer's disease at a younger age and have shorter lifespans, highlighting the impact of psychiatric comorbidity on longevity.

## 1 Introduction

Psychiatric symptoms and disorders are prevalent in people with Alzheimer's disease (AD), often emerging years or even decades before AD diagnosis [1, 2, 3]. Psychiatric disorders have a significant impact on quality of life. Despite this, their impact on mortality in people with AD remains unclear due to the limited number of studies [4, 5, 6, 7]; [8].

Furthermore, these previous studies have focused mainly on depression or depressive symptoms after AD/dementia diagnosis [4, 5, 6, 7]. Found that a history of any psychiatric disorder before diagnosis of AD was associated with increased mortality, but did not analyze chronic and more recent diagnoses separately [8]. Thus, there are no previous studies analyzing mortality in AD based on a history of psychiatric morbidity. Most of the above‐mentioned studies on incident depression or depressive symptoms reported higher mortality among those with both depression and AD, compared to those with AD but without depression [4, 5, 7], while one did not observe a difference in mortality [6]; however, the timing of depression diagnosis or depressive symptoms in relation to AD/dementia diagnosis was unclear in that study. Therefore, those studies may be more representative of the impact of prodromal symptoms or neuropsychiatric symptoms of AD rather than actual psychiatric comorbidity. Thus, little is known about how psychiatric disorders diagnosed in different time windows relate to the mortality of people with AD.

We have previously identified four distinct groups based on the time of occurrence of psychiatric diagnoses that required specialized healthcare, in a nationwide cohort of people with a clinically verified AD diagnosis [9]. This allowed us to investigate Alzheimer's disease mortality based on the timing of earlier psychiatric morbidity spanning up to 30 years before AD diagnosis, with follow‐up data spanning up to 8 years. However, it is important to note that in the treatment of Alzheimer's disease, maintaining a good quality of life is often more meaningful than merely extending life expectancy. Nonetheless, investigating this relationship is essential, as psychiatric morbidity may affect not only survival but also the quality of life of individuals with AD, as well as that of their families and caregivers.

### 1.1 Aims

This study aimed to compare mortality and causes of death across four groups categorized by the timing of psychiatric morbidity before Alzheimer's disease diagnosis, to clarify how earlier diagnosed psychiatric disorders relate to mortality in AD.

## 2 Materials and Methods

### 2.1 Medication Use and Alzheimer's Disease (MEDALZ) Cohort

The MEDALZ study is a Finnish nationwide register‐based cohort including 70,718 individuals diagnosed with Alzheimer's disease in Finland between 2005 and 2011 who were community‐dwelling at the time of diagnosis. Their AD diagnosis was clinically verified using NINCDS‐ADRDA and DSM‐IV criteria, CT/MRI scans, differential diagnosis between other diseases, especially neurodegenerative diseases, and confirmation by a neurologist or geriatrician. The study retrieved morbidity and mortality data from the following national registers: the National Institute for Health and Welfare Care Register for Health Care (diagnoses of psychiatric disorders and somatic illnesses, hospitalizations 1972–2011), Social Insurance Institution special reimbursement register (chronic comorbidities; 1972–2011), and Statistics Finland cause of death register (2005–2019). The study cohort has been described previously [10].

### 2.2 The Study Population, Follow‐Up, and Mortality

For this study, the cohort members were categorized into four groups based on their history of psychiatric diagnoses treated in specialized healthcare, excluding primary healthcare psychiatric diagnoses, as the cohort data does not include information on primary care diagnoses. The groups were as follows: no psychiatric diagnosis in specialized healthcare before diagnosis of AD, (*n* = 64,703), previous diagnosis (psychiatric diagnosis more than 5 years before AD, *n* = 3009), prodromal (psychiatric diagnosis up to five years before AD, *n* = 2222), and chronic (psychiatric diagnosis both less than and more than 5 years before AD, *n* = 784). Psychiatric diagnosis during a 5‐year time window before the diagnosis of Alzheimer's disease was selected as the definition of the prodromal group based on clinical experience and previous evidence [2, 3, 11]. In our previous study, we reported the distribution of psychiatric diagnoses in this cohort: 19% had schizophrenia, delusional disorder, or related psychoses; 21% had other psychotic disorders; 4% had non‐psychotic bipolar disorder; 41% had depression; and the remaining participants had other psychiatric disorders [9].

The follow‐up began on the date of AD diagnosis and ended on death or after 8 years of follow‐up. In addition to all‐cause mortality, we assessed the proportion of falls (ICD‐10: W00‐W19), hip fractures (S720‐S722), traumatic brain injuries (S06‐S07), dementia or Alzheimer's disease (F00‐F03, G30), vascular diseases (I00‐I425, I427‐I99), strokes (I60‐I64, I69), alcohol‐related deaths (F10, G312, G4051, G621, G721, I426, K292, K70, K852, K860, X45), poisoning (excluding alcohol poisoning, X40‐X44, X46‐X49, Y10‐Y19) and suicides (X60‐X84, Y87.0) as causes of death. Only the underlying cause of death was considered in assigning the causes of death in this study, as we wanted to avoid double‐counting. Dementia or Alzheimer's disease as the cause of death was therefore included for analysis, as clinical experience suggests that death in these individuals more often results from a long‐term gradual decline in functional capacity rather than another acute underlying illness. Consequently, this is typically recorded as the underlying cause of death on the death certificate. We were particularly interested in alcohol‐related deaths and accidental deaths because in our previous study, substance abuse was strongly connected with belonging to the prodromal group (RR 65.1, 95% CI 55.5–76.2) [9].

Mortality was also examined according to categories of psychiatric diagnoses. Psychiatric disorders were classified into hierarchical groups using ICD‐8, ICD‐9, and ICD‐10 codes. The schizophrenia spectrum group included schizophrenia and related disorders (ICD‐10: F20, F22, F24, F25; ICD‐9: 2950–2959, 297; ICD‐8: 2950–2959, 297). The group of other psychotic and bipolar disorders comprised bipolar disorder and non‐schizophrenic psychotic disorders (ICD‐10: F23, F28, F29, F300–F301, F302, F303–F311, F312, F313–F314, F315, F316–F319, F323, F333; ICD‐9: 2961E, 2962A–D, 2962E–G, 2963A–D, 2963E–G, 2964A–D, 2964E–G, 2967–2969, 2988–2989; ICD‐8: 2960–2969, 2980–2983, 2988–2989, 299). Other psychiatric disorders included depressive, anxiety, and other psychiatric diagnoses (excluding those mentioned above; ICD‐10: F10–F69, F90–F99, ICD‐9: 295–298, 300–301, 3004, 3071–3075, 312–314; ICD‐8: 295–301, 303–308, 7902). For individuals with multiple psychiatric diagnoses, only the most severe diagnosis was considered in the analyses.

### 2.3 Statistical Analysis

Cross‐tabulation and the Chi‐square test were used to compare the differences between the four groups in terms of causes of death and annual cumulative mortality. We determined cumulative mortality curves and hazard ratios, stratified by age, sex, cardiovascular diseases (essential hypertension: I10–I13, I15, I27.0, I27.2, coronary artery disease: I20–I22, I24.0, I25, heart failure: I11.0, I13, I50, I97.1, P29.0), and year of AD diagnosis. Statistical analyses were conducted using IBM SPSS Statistics.

## 3 Results

The no diagnosis group had the highest age at the time of AD diagnosis (80.2 years), and the chronic group the lowest (75.9 years) [Table 1]. The prodromal group had a higher proportion (69.2%) of women than the other groups (65.0%–67.7%).

**TABLE 1:** Characteristics and mortality of the sample.

Variable	No diagnosis (*n* = 64,703)	Previous diagnosis (*n* = 3009)	Prodromal (*n* = 2222)	Chronic (*n* = 784)	*p*‐value
Sex, *n* (%)					< 0.001
Women	42,069 (65.0)	1979 (65.8)	1537 (69.2)	531 (67.7)	
Men	22,634 (35.0)	1030 (34.2)	685 (30.8)	252 (32.3)	
Age at AD diagnosis, mean (95% Cl)	80.2 (80.2–80.3)	78.8 (78.5–79.0)	78.6 (78.3–78.9)	75.9 (75.3–76.4)	< 0.001
Women	80.9 (80.8–80.9)	79.9 (79.6–80.2)	79.7 (79.3–80.0)	77.0 (76.3–77.7)	< 0.001
Men	79.0 (78.9–79.1)	76.7 (76.3–77.1)	76.2 (75.6–76.8)	73.5 (72.5–74.4)	< 0.001
Age at death, mean (95% CI)	85.7 (85.6–85.8)	84.3 (84.0–84.6)	83.9 (83.6–84.3)	81.6 (80.9–82.3)	< 0.001
Women	86.7 (86.7–86.8)	85.7 (85.4–86.1)	85.3 (84.9–85.7)	83.3 (82.5–84.1)	< 0.001
Men	84.0 (83.9–84.1)	81.8 (81.4–82.3)	81.2 (80.5–81.8)	78.3 (77.2–79.5)	< 0.001
Cumulative mortality, *n* (%)					
Within 1 year of AD diagnosis	3794 (5.9%)	199 (6.6%)	135 (6.1%)	45 (5.7%)	0.381
Within 2 years of AD diagnosis	9241 (14.3%)	436 (14.5%)	338 (15.2%)	93 (11.9%)	0.144
Within 3 years of AD diagnosis	15,568 (24.1%)	725 (24.1%)	574 (25.8%)	161 (20.5%)	0.028
Within 4 years of AD diagnosis	22,232 (34.4%)	1022 (34.0%)	781 (35.1%)	240 (30.6%)	0.129
Within 5 years of AD diagnosis	28,995 (44.8%)	1294 (43.0%)	995 (44.8%)	312 (39.8%)	0.010
Within 6 years of AD diagnosis	35,145 (54.3%)	1570 (52.2%)	1196 (53.8%)	383 (48.9%)	0.002
Within 7 years of AD diagnosis	40,859 (63.1%)	1833 (60.9%)	1373 (61.8%)	439 (56.0%)	< 0.001
Within 8 years of AD diagnosis	45,686 (70.6%)	2054 (68.3%)	1544 (69.5%)	494 (63.0%)	< 0.001
Follow‐up length in years, median (IQR)	5.53 (3.10–8.00)	5.75 (3.04–8.00)	5.53 (2.94–8.00)	6.21 (3.41–8.00)	0.381
Living years after diagnosis of AD^a^, mean (SD)	4.10 (2.13)	4.06 (2.17)	4.02 (2.15)	4.13 (2.13)	0.430

Abbreviations: AD, Alzheimer's disease; CI, Confidence interval; IQR, Interquartile range; SD, Standard deviation.

^a^ Variable includes only persons who have died during the follow‐up period.

Median follow‐up length was 5.53 years in the no diagnosis group and prodromal group, 5.75 years in the previous diagnosis group, and 6.21 years in the chronic group [Table 1]. By the end of the 8‐year follow‐up period, 70.4% of the study population had died. Mean survival after AD diagnosis ranged from 4.02 to 4.13 years among those who died during the follow‐up and did not differ between the four groups. The mean age at death was the highest in the no diagnosis group and the lowest in the chronic group. The no diagnosis group had the highest proportion of deaths (70.6%), while the chronic group had the lowest (63.0%).

**FIGURE 1:**
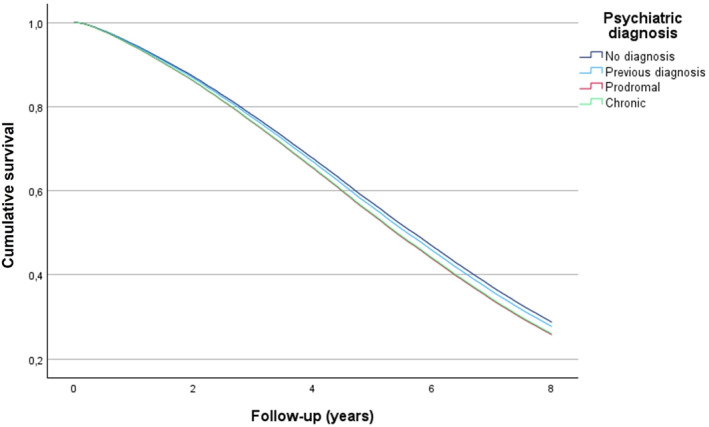
Cumulative survival, adjusted with age, sex, vascular diseases, and year of AD‐diagnosis. *The chronic and prodromal groups overlap in the lowest curve.

In crude analyses, compared to the no diagnosis group, the chronic group showed statistically significantly lower unadjusted hazard ratios at years 3, 5, and 8. Still, this association was lost when adjusted by age at the AD diagnosis, sex, cardiovascular diseases, and year of AD diagnosis, of which age at the AD diagnosis was the strongest adjuster, while others had only a marginal effect [Table 2, Figure 1]. In adjusted analyses, the no diagnosis group had statistically significantly lower mortality than any other group for the first 5 years after AD diagnosis. There were no significant differences in mortality risk between previous‐, prodromal‐, and chronic psychiatric diagnosis groups.

**TABLE 2:** Hazard ratios for death (The no diagnosis group is the reference).

	Unadjusted		Adjusted^a^	
	HR (95% CI)	*p*‐value	HR (95% CI)	*p*‐value
1 year	1.00 (reference)	0.419	1.00 (reference)	< 0.001
Previous	1.13 (0.98–1.30)		1.27 (1.10–1.46)	
Prodromal	1.04 (0.88–1.23)		1.19 (1.01–1.42)	
Chronic	0.98 (0.73–1.31)		1.41 (1.05–1.90)	
3 years	1.00 (reference)	0.033	1.00 (reference)	< 0.001
Previous	1.00 (0.93–1.08)		1.12 (1.04–1.21)	
Prodromal	1.08 (0.99–1.18)		1.23 (1.13–1.33)	
Chronic	0.84 (0.72–0.98)		1.18 (1.01–1.38)	
5 years	1.00 (reference)	0.017	1.00 (reference)	< 0.001
Previous	0.96 (0.90–1.01)		1.05 (1.00–1.11)	
Prodromal	1.01 (0.95–1.08)		1.13 (1.06–1.20)	
Chronic	0.85 (0.76–0.95)		1.16 (1.04–1.30)	
8 years	1.00 (reference)	< 0.001	1.00 (reference)	0.002
Previous	0.95 (0.91–0.99)		1.03 (0.99–1.08)	
Prodromal	0.98 (0.94–1.03)		1.09 (1.03–1.14)	
Chronic	0.82 (0.75–0.90)		1.8 (0.99–1.18)	

Abbreviations: Chronic, present both > 5 years before and within 5 years before AD diagnosis; CI, Confidence interval; HR, Hazard ratio; Previous, more than 5 years before diagnosis of AD; Prodromal, within 5 years before diagnosis of AD.

^a^ Age, sex, cardiovascular disease, and year of AD diagnosis.

When mortality was analyzed according to type and timing of psychiatric diagnoses, modest but statistically significant differences were observed. Chronic schizophrenia spectrum disorder (occurring both less than and more than 5 years before AD) was associated with higher mortality. In contrast, other psychiatric diagnoses were associated with increased mortality only when they occurred within 5 years before the Alzheimer's disease diagnosis [Table 3].

**TABLE 3:** Hazard ratios for mortality during follow‐up by history of psychiatric diagnoses, considering the most severe psychiatric disorder per individual (The no diagnosis group is the reference).

	Adjusted^a^	
	HR (95% CI)	*p*‐value
Schizophrenia spectrum	1.00 (reference)	
Previous	1.04 (0.93–1.17)	0.521
Prodromal	1.04 (0.96–1.18)	0.249
Chronic	1.27 (1.05–1.54)	0.014
Other psychosis or bipolar disorder	1.00 (reference)	
Previous	1.06 (0.96–1.17)	0.263
Prodromal	1.16 (1.04–1.30)	0.011
Chronic	1.08 (0.83–1.39)	0.577
Other psychiatric disorder	1.00 (reference)	
Previous	1.02 (0.97–1.08)	0.408
Prodromal	1.07 (1.00–1.14)	0.040
Chronic	1.02 (0.88–1.17)	0.806

Abbreviations: Chronic, present both > 5 years before and within 5 years before AD diagnosis; CI, Confidence interval; HR, Hazard ratio; Previous, more than 5 years before diagnosis of AD; Prodromal, within 5 years before diagnosis of AD.

^a^ Age, sex, cardiovascular disease, and year of AD diagnosis.

Dementia or AD and vascular diseases were the most common causes of death in all four groups [Table 4], with dementia or AD being the most common in the no diagnosis group (48.5% of all causes of death) and the least common in the chronic group (42.1%). The proportion of alcohol‐related deaths was the highest in the chronic group (1.0%) and the prodromal Group (0.8%). There were no statistically significant differences between the groups in falls, poisoning, or cardiovascular diseases as causes of death.

**TABLE 4:** Causes of death (follow‐up period from AD diagnosis to death or 8 years).

Variable	No diagnosis (*n* = 64,703)	Previous diagnosis (*n* = 3009)	Prodromal (*n* = 2222)	Chronic (*n* = 784)	*p*‐value
Any	45,686	2054	1544	494	
Dementia or Alzheimer's disease (% of deaths)	22,153 (48.5%)	865 (42.1%)	698 (45.2%)	216 (43.7%)	< 0.001
Vascular diseases	14,803 (32.4%)	715 (34.8%)	518 (33.5%)	164 (33.2%)	0.110
Stroke	3478 (7.6%)	161 (7.8%)	127 (8.2%)	29 (5.9%)	0.378
Falls	1173 (2.6%)	44 (2.1%)	39 (2.5%)	17 (3.4%)	0.390
Hip fracture	579 (1.3%)	21 (1.0%)	20 (1.3%)	6 (1.2%)	0.806
Traumatic brain injury	420 (0.9%)	16 (0.8%)	11 (0.7%)	6 (1.2%)	0.660
Alcohol‐related deaths	33 (0.1%)	5 (0.2%)	12 (0.8%)	5 (1.0%)	< 0.001
Poisoning	11 (0.0%)	< 5 (0.2%)	0 (0%)	< 5 (< 0.6%)	0.188
Suicides	33 (0.1%)	< 5 (< 0.2%)	< 5 (< 0.2%)	< 5 (< 0.6%)	0.006

## 4 Discussion

### 4.1 Main Findings

Our study found that individuals with AD and hospitalization due to a psychiatric disorder before AD had a shorter overall lifespan compared to those without such a history. They were also diagnosed with AD at a younger age than those without psychiatric morbidity. There were no large‐scale differences in mortality risk after AD diagnosis between previous, prodromal, and chronic groups, although all psychiatric comorbidity groups had a slightly higher risk of death compared to the no diagnosis group. The most common causes of death were dementia/AD and vascular diseases in all groups. Alcohol‐related deaths were more common in the chronic and the prodromal groups. We cautiously suggest that we have identified a subgroup of Alzheimer's disease that has a slightly worse prognosis and is more likely to have a history of psychiatric disorders and substance abuse in the years preceding AD diagnosis. This subgroup could be one to focus on health policy to improve not only prognosis, but especially quality of life.

### 4.2 Comparison to Previous Studies

Previous studies have mainly investigated depressive symptoms and their impact on the prognosis of Alzheimer's disease. However, it is known that depressive symptoms emerging at the time of or shortly before an Alzheimer's disease diagnosis are most likely prodromal symptoms of AD. Some studies have reported an association between depression or depressive symptoms and higher mortality in Alzheimer's disease or dementia [4, 5, 7, 12]. However, some of these studies have had challenges with depression diagnostic methods, and there has been confusion with the concepts (depressive symptoms vs. depression), and it has not been revealed whether depressive symptoms or the depression diagnosis occurred before or after the AD diagnosis. Zivin et al. found in their retrospective population‐based cohort study (*N* = 5,078,082) of the association between depression and all‐cause and cause‐specific risk of death, that depression was associated with higher 3‐year mortality in Alzheimer's disease (HR 1.61, 95% CI). However, the study did not report how the diagnosis of depression was timed in relation to Alzheimer's disease [12]. In the same study population as the present study, any mental or behavioral disorder before AD diagnosis was associated with increased mortality risk among persons with AD (adjusted HR 1.11, 95% CI) [8]. In a small sample of home‐dwelling persons with recently diagnosed dementia, mainly AD, persons with depressive symptoms were at increased risk of death (HR 2.55, 95% CI) [7]. In the study of predictors of mortality in people with dementia, depressive symptoms (evaluated with a geriatric depression scale, but without defining which GDS questionnaire was used) were associated with a very slight increase in mortality of AD (HR 1.045, 95% CI) [4]. Lara et al. found that self‐reported depressive symptoms increased mortality in incident Alzheimer's disease (HR 1.88, 95% CI), though methodological limitations, such as reliance on self‐reported data of depressive symptoms at one time‐point, temper the findings [5]. Perna et al. conducted a longitudinal cohort study to investigate the effect of incident depression on the mortality of incident dementia. There were no statistically significant differences in mortality risk in persons with Alzheimer's disease, whether depression occurred or not [6]. The study did not define when depression occurred, which reduces the relevance of the result.

Although previous studies are not fully comparable with our study, and the HRs in our study are slightly lower, it can also be concluded from our study that psychiatric morbidity slightly worsens the prognosis of Alzheimer's disease. In our study, approximately 30% of the individuals were still alive at the end of the 8‐year follow‐up period. On average, the mean survival among those who died was 5.5 years. There are no major differences in life expectancy after an AD diagnosis between the groups, suggesting that AD seems to override the impact of many other diseases on life expectancy. The type and timing of psychiatric disorders had an impact on mortality, especially chronic schizophrenia spectrum disorders, but the differences were quite small. The lower mortality risk in groups with psychiatric comorbidity in unadjusted analyses seems to be explained by their younger age at AD diagnosis, because after adjusting for age, higher mortality was observed in the psychiatric comorbidity groups compared with the no psychiatric diagnosis group. However, the differences were small. It is plausible that the neurodegeneration and functional decline associated with Alzheimer's disease have such a profound impact on lifespan that coexisting psychiatric illnesses do not substantially alter the course of the disease.

In addition to psychiatric illness and symptoms [4, 5, 7]; [8]; [12], somatic comorbidities can shorten life expectancy. As reported in our previous study, individuals in the no diagnosis group had fewer comorbidities, such as asthma/COPD, cardiovascular diseases, and diabetes [9]. However, in the present study, the adjustment for other comorbidities, sex, or year of AD diagnosis had only a minimal impact on the HRs, and age was the most influential adjustment factor.

Several factors could explain why people with psychiatric disorders receive an earlier diagnosis of AD and why they die earlier. Psychiatric disorders are associated with the process of neurodegeneration, functional decline, and social isolation that can predispose to cognitive decline and an earlier diagnosis of AD. The high prevalence of metabolic disorders, such as diabetes mellitus, in people with psychiatric morbidities can also increase the risk of cognitive disorders [13, 14, 15, 16, 17, 18].

The most common reasons for death in this study were dementia/AD and vascular diseases, which were expected. Alzheimer's disease and other cognitive disorders are associated with an increased risk of suicide, and the risk is increased especially in the early stages of diagnosis [19, 20, 21]. On the other hand, it might be that the risk of suicide has not increased among the population with Alzheimer's disease, but the risk of accidental death has increased [22]. During the years 2011–2019, suicide was the cause of death in 0.29% (1123/492,076) of all deaths in people over 75 years old [23]. In our dataset, suicide accounted for a lower proportion (< 0.09%; < 48/49,778) of deaths. Hence, the number of suicides in this study does not substantially differ from the general population with the same age group. On the other hand, there may be challenges in identifying suicides in persons with cognitive disorders, and they may be assigned as accidental deaths instead. In many cases, the circumstances of accidents involving people with dementia may also be unclear. So, there may have been more suicides than reported in the causes of death register. There was a suggestion of a higher rate of alcohol‐related deaths in the prodromal and chronic groups, although the differences were not clinically significant. According to Statistics Finland, among individuals aged over 70 years during 2011–2020, alcohol‐related deaths accounted for 0.58%–0.90% of all deaths when considering the underlying cause of death. The corresponding proportions were higher among men (1.04%–1.51%) than among women (0.23%–0.42%) [23]. Compared to this, there were fewer deaths in our data. Together with our previous finding of more prevalent substance abuse in the prodromal group [9], we suggest that attention is paid to alcohol use in the clinical management of Alzheimer's disease, while also addressing other mental health conditions.

## 5 Strengths and Limitations

The history of hospital‐treated psychiatric morbidity and its association with mortality in AD has not been previously studied in a prospective, unselected cohort. None of the previous studies examined the history of psychiatric morbidity with as long lookback period as applied in this study, accounting for diagnoses even from decades ago. Previous studies have primarily focused on incident depression/depressive symptoms. More specifically, previous studies have not necessarily clearly shown how the depression diagnosis was defined, whether it was a depressive symptom, or at what time these diagnoses or symptoms appeared in relation to the Alzheimer's disease diagnosis, whereas we were able to analyze the association between psychiatric disorders occurring at different periods of life and mortality in AD. Furthermore, our study specifically included diagnosed psychiatric illnesses, not just symptoms.

In general, Finnish health registers are known for their high coverage and accuracy [24, 25]. This study leverages a large, nationwide cohort, which enhances the generalizability of our findings. The use of comprehensive, register‐based data from multiple national sources enables the identification of psychiatric comorbidities treated in specialized healthcare, as well as mortality and causes of death, over an extended follow‐up period of up to 8 years. By categorizing individuals based on detailed psychiatric history and adjusting for multiple covariates, our study provides a nuanced understanding of how psychiatric comorbidities may impact mortality in Alzheimer's disease. Despite the strengths, several limitations exist, including that less severe psychiatric disorders that are treated outside psychiatric hospitals or in primary care are not included in our data. The study also lacks detailed clinical information, for example, on the severity of Alzheimer's disease at the time of diagnosis. The information on psychiatric diagnoses is based on the national register of psychiatric disorders treated in hospitals, which may underestimate psychiatric morbidity, particularly less severe disorders or those treated in outpatient settings. Also, changes in psychiatric inpatient care practices over time, including a significant reduction in psychiatric hospital beds in recent decades, may have affected the recording and timing of psychiatric diagnoses, which may affect the interpretation of hospitalization data in our data.

## 6 Conclusion

Individuals with AD who had been hospitalized due to a psychiatric disorder before AD had a shorter overall lifespan compared to those without such a history. There were no significant differences in survival time after AD diagnosis, but people with psychiatric comorbidity were diagnosed with AD at a younger age. Thus, while psychiatric comorbidity is associated with earlier AD onset and reduced lifespan, post‐diagnosis survival seems to be largely determined by AD progression itself.

## Funding

This work was supported by a grant from the Academy of Finland (Grant No. 316563), Oulu University Hospital funding (basic government funding for hospitals), and Eka Grant from the Finnish Medical Foundation. The funders had no role in the study design, data collection, data analysis, interpretation of the results, or the decision to publish the article.

## Ethics Statement

According to Finnish legislation, ethics committee approval or individual consents are not needed for solely register‐based studies. The MEDALZ study protocol has been approved by the relevant keepers of the registers. The study has been performed in accordance with the ethical standards laid down in the 1964 Declaration of Helsinki and its later amendments.

## Conflicts of Interest

The authors declare no conflicts of interest.

## Data Availability

The data that support the findings of this study are available from the corresponding author, but restrictions apply to the availability of these data, and so the data are not publicly available. Requests regarding the data can be sent to the authors and the register maintainers.
